# New Appliances and Things Medical

**Published:** 1897-06-26

**Authors:** 


### NEW APPLIANCES AND THINGS MEDICAL.

[We shal be glad to receive, at our Office, 28 & 29, Southampton Street, Strand, London, W.C., from the manufacturers, specimens of all new preparations and appliances which may be brought out from time to time.

### GENEVA MINERAL WATER.

(Agents, 10a, Green Street, Leicester Square, London, W.C.)

The success which has attended the introduction of the continental mineral waters to this country has stimulated the United States to imitate the foreign bottlers. This Geneva Mineral Water is imported into this country from Geneva, New York, where a mineral spring exists, the waters from which have already been largely consumed in America. The water is recommended as a regulator, tonic and alterative, and is stated to contain phosphoric acid, iron, chloride of sodium, sulphate of soda, chloride of potassium, sulphate of magnesium, carbonate and sulphate of lime, carbonate of magnesia and lithia. Although a full analysis of the water is not given by the proprietors, it is obvious that a water containing these ingredients in fair proportion would have a certain medicinal value, and could be used with advantage in cases in which a mild, alterative treatment was desired. We instructed our analyst to make a partial analysis of the samples of water sent to us for examination with the view to ascertain whether the above ingredients were present, and for his opinion as to the remedial nature of the water. In his report he states that the total solids amount to 319·20 parts per 100,000, showing that the water is very rich in saline constituents, and his further examination shows that the water is practically free from organic matter or other injurious constituents. The approximate analysis in parts per 100,000 is as follows:—

| | |
|---|---|
| Chlorine | 19·79 |
| = as Sodium Chloride | 32·61 |
| $SO_3$ | 127·49 |
| Ca O | 78·62 |
| Mg O | 17·03 |
| $CO_2$ | 9·13 |
| Alkalies (by difference) | 67·14 |

The water was clear and free from any appreciable colour. It contained no sediment, but contained a slight trace of iron. There was a distinct trace of phosphate, but it is difficult to understand how any amount of phosphate of iron can exist in such a water. The analysis shows that the water belongs to the class of saline mineral waters containing a notable amount of magnesium salts, and that consequently it acts as a mild aperient or regulator. Six to eight glasses a day is the quantity prescribed on the bottle to effect a cure (it does not say from what), whilst for "regulation drink" only two or three glasses are required. The water has a pleasant saline taste, and is very agreeable to the palate, although it does not possess any appreciable amount of free carbonic acid. We recommend a trial of the water to those who are in need of a mild aperient water, and who find the stronger magnesian waters too powerful.

### TUBERCULOCIDIN (KLEBS).

(Ferris and Co., Bristol.)

We have been supplied with samples of the above serum remedy, which, like its congener antiphthisin, is a remedy which differs in principle from the usual anti-toxins. It is made from a sterilised culture of the tubercle bacilli, and may be injected subcutaneously or per rectum. Unlike the anti-toxins it is slow in its action, and a course of treatment to be effective must extend over many months or even years, periods of rest intervening between the periods of active treatment. There appear reasons for believing that there are considerable merits in this new serum preparation, but to pronounce a definite opinion is impossible until many new investigations have been made by independent observers. The treatment is one that can be carried out efficiently in private practice and apparently without danger. Practitioners in this country have therefore a favourable opportunity of adding to the world's knowledge by independent research, which can be carried on with no excessive demand on their time or energies.

### PHŒNIX DIGESTIVE FOOD.

(Phœnix Food Co., 39, Exchequer Street, Dublin.)

This digestive food, when made according to the directions supplied, constitutes an agreeable and nutritious food for invalids and young children. For infants during the transitional stage between exclusive milk diet and the period when with the eruption of the milk teeth a more solid dietary is indicated, we believe that the Phœnix digestive food is peculiarly applicable. It appears to be well tolerated by invalids whose ailments are chiefly due to impaired powers of digestion.

